# Tumor Heterogeneity in Primary Colorectal Cancer and Corresponding Metastases. Does the Apple Fall Far From the Tree?

**DOI:** 10.3389/fmed.2018.00234

**Published:** 2018-08-31

**Authors:** Annika Blank, Daniel Edward Roberts, Heather Dawson, Inti Zlobec, Alessandro Lugli

**Affiliations:** ^1^Department of Pathology, University of California, San Francisco, San Francisco, CA, United States; ^2^Clinical Pathology Division, Institute of Pathology, University of Bern, Bern, Switzerland; ^3^Translational Research Unit, Institute of Pathology, University of Bern, Bern, Switzerland

**Keywords:** heterogeneity, colorectal cancer, metastases, clonal evolution, cancer stem cells

## Abstract

Colorectal cancer harbors tremendous heterogeneity, with temporal and spatial differences in genetic mutations, epigenetic regulation, and tumor microenvironment. Analyzing the distribution and frequency of genetic, epigenetic, and microenvironment differences within a given tumor and between different sites of a metastatic tumor has been used as a powerful tool to investigate tumorigenesis, tumor progression, and to yield insight into various models of tumor development. A better understanding of tumor heterogeneity would have tremendous clinical relevance, which may manifest most clearly when genetic analyses to inform treatment decisions are performed on a very limited sample of a large tumor. This review summarizes the current concepts of tumor heterogeneity, with a focus on primary colorectal cancers and their corresponding metastases as well as potential clinical implications.

## Colorectal cancer is not just colorectal cancer

The last two decades have seen emerging interest and increasing awareness in tumor heterogeneity, a consequence of tumorigenesis that is relevant to the study of many malignant neoplasms [1].

In particular, colorectal cancer (CRC) is known to harbor considerable heterogeneity [2]. By comparing microsatellite stable and unstable CRC, it has been described that the presence of various growth patterns within a tumor is significantly more common in microsatellite unstable tumors [3, 4]. Yet it has also been shown that morphologic differences do not necessarily indicate genetic differences, and vice-versa [5]. Consequently, morphologically diverse areas within a tumor may have a similar genetic landscape, while similar appearing tumor fractions might exhibit substantial genetic differences. This phenomenon is caused by non-genetic influences such as epigenetic regulation, post-translational modifications, or a differential tumor microenvironment. This review summarizes our current understanding of tumor heterogeneity in CRC and corresponding metastases, beginning with the basic genetic background, alterations in RNA and protein expression, and finally the impact of tumor microenvironment beyond the cancer cell itself. Intra-tumoral heterogeneity is an additional important aspect to be considered but is beyond the scope of this review. One example is the potential heterogeneous expression of mismatch-repair proteins (MLH1, MSH2, MSH6, and PMS2) [6] as the mismatch-repair status seems to play a role in terms of a chemotherapy and immunotherapy [7].

## Heterogeneity through time and space

The concept of inter-tumor heterogeneity refers to differences either between synchronous primary tumors of the same type developing in the same patient, or between a primary tumor and its matched metastases. In contrast, intra-tumor heterogeneity refers to differences within the same neoplasm. Heterogeneity can be then further sub-classified as spatial or temporal, where spatial heterogeneity describes variations in distinct regions of a tumor, and temporal heterogeneity refers to differences that develop within a given tumor over during time.

## The genesis of tumor heterogeneity—current concepts

Various models have been proposed to describe the origin and role of tumor heterogeneity. Is tumor heterogeneity a *conditio sine qua non*, or merely a byproduct of tumorigenesis and tumor progression? Different concepts of tumorigenesis offer different answers.

### The cancer stem cell model

The cancer stem cell model posits that only a minority of cells in a given tumor are capable of tumor initiation and progression. These so-called cancer stem cells represent the basic unit of tumor growth and metastatic spread, while the majority of any given neoplasm consists of non-tumorigenic cells incapable of metastatic seeding or tumor progression.

These tumor initiating cells have been described in the literature through specific surface proteins, such as CD133, CD166, or CD44 [8–10], which on average account for 11.4% of epithelial cells in primary CRC [10]. Subcutaneous injection of human CD133^+^ tumor cells into immune-deficient mice led to the formation of tumors with morphologic similarity to the original neoplasm, even after as much as 1 year of growth *in vitro*. In contrast, no tumor formation was seen following injection of CD133^−^ tumor cells alone [8]. Even after serial transplantation in xenograft mice, CD133^+^ cells initiated tumors phenotypically identical to the initial CRC [9].

Dylla et al. [11] demonstrated that the cancer stem cell fraction is increased in tumors after chemotherapy, and may help explain relapse following treatment. Perhaps related, interleukin 4 is produced by cancer stem cells, and has previously been shown by Todaro et al. to foster anti-apoptotic pathways in stem cells, causing reduced sensitivity to chemotherapy [12].

### Model of clonal evolution

A contrasting concept follows a Darwinian approach, proposing that cancer development proceeds by branching, clonal evolution. In this model, the stepwise acquisition of mutations or non-genetic alterations yields tumor sub-clones with varying capacity to adapt to the tumor microenvironment, therefore leading to heterogeneity within and between tumors.

This would imply that evolutionary branching can be tracked by analyzing the distribution and frequency of mutations in a tumor. Ubiquitous mutations that are present in all tumor cells are likely to represent early events in tumor development. Conversely, the later a mutation occurs during tumor progression, the less likely is it to be found in other tumor sub-clones. Conceptually, ubiquitous mutations would represent the trunk of the Darwinian tree (the so-called trunk mutations), and the branches would be represented by later mutational events (so called branching mutations) [13, 14]. Yachida et al. demonstrated the presence of these two different mutational groups in pancreatic adenocarcinomas and their corresponding metastases. Trunk mutations constituted the majority of mutations (64%) in both primary and metastatic tumors and may represent early events in tumorigenesis. In addition, the authors showed that all metastatic sub-clones could be identified in the primary tumor [15].

Gerlinger et al. supported the concept of branching evolution in clear cell renal cancer by phylogenetic reconstruction based on the mutation pattern [16]. Others have shown that CRC and a subset of breast cancer seem to follow the clonal evolution concept [5, 17].

To investigate heterogeneity in different stages of tumor progression, Losi et al. [18] analyzed KRAS, p53 mutations, and loss of heterozygosity on chromosome 5 and 18q in different areas of primary CRC lesions as well as their corresponding metastases. Surprisingly, point mutations decreased with tumor progression, and loss of heterozygosity tended to be stable throughout progression. Advanced tumors often had one dominant clone with multiple minor sub-clones, whereas a dominant clone was found less frequently in early stage CRC. In distant metastases, tumor heterogeneity for KRAS and p53 mutations was rare. This observation of decreasing tumor heterogeneity with tumor progression, especially in distant metastases, is supported by Kim et al, who demonstrated a higher frequency of mutated alleles in CRC metastases compared to matched primaries, pointing toward a narrowing in genetic and non-genetic heterogeneity in corresponding metastases. This phenomenon was termed “bottleneck effect” and refers to an at least temporary decrease in heterogeneity due to selection of sub-clones during metastatic seeding [17]. Tumor therapy is a second substantial bottleneck for tumor progression [13, 19]. Due to these bottleneck events, branch mutations might become important for the tumor in order to adapt to new conditions. Yap et al. hypothesized that the longer the trunk and the less branching a tumor has, the more likely is it to find a targetable mutations with high therapy response rates. Conversely, tumors with pronounced branching are more likely to harbor a therapy-resistant sub-clone as a consequence of increased genetic diversity. Based on this assumption, the authors proposed the degree of tumor heterogeneity itself as a potential biomarker [13]. Indeed, an association between increased tumor heterogeneity and poor prognosis was shown in esophageal and breast cancer [20, 21].

### The big bang model

Sottoriva et al. [22] suggested a different view of heterogeneity by emphasizing the temporal aspect of tumor mutations. This “big bang model” hypothesizes that the mutations responsible for tumor development and progression occur early in CRC. The biological behavior of a tumor is therefore determined early, which may explain why some large tumors never metastasize, and some small tumors develop early metastases (“born to be bad”). Single gland analysis in different tumor areas was used to map the regional distribution of genetic alterations in CRC. Separated sub-clones could only be identified in adenomas, whereas merging sub-clones were only a characteristic of invasive carcinomas. This spatial and temporal analysis would support a single clonal expansion concept. Rather than dominant sub-clones spatially overgrowing others, they found extensive mixing of sub-clones. In this model, additional mutations that give rise to different tumor sub-clones could therefore be considered bystander mutations rather than driven by Darwinian selection of the “fittest” sub-clone that leads to spatial dominance.

All these concepts of tumorigenesis have been supported with compelling data. It is likely that they represent complementary models rather than mutually exclusive ones. The identification of cancer stem cells, for example, is primarily based on expression of surface proteins [23], whereas the clonal evolution [24] and big bang models are based on genetic analysis. Bottleneck conditions might influence tumor dynamics with selection and overgrowth of the fittest and best adapted sub-clone for the given circumstances [2].

## From models to observations—genetic heterogeneity in primary colorectal cancer and corresponding metastases

Although the origin of tumor heterogeneity is not fully understood, its existence is well-known both in primary CRC and corresponding metastases.

The genetic landscape of CRC consists of several high mountains, representing common and well-known mutations, with innumerable small hills, representing non-recurrent mutations. An average CRC harbors ~ 80 mutations, yet fewer than 15 mutations seem to be the driving force for tumorigenesis and progression. Interestingly, the mutational profile of any two CRC primaries shows minimal overlap, and the vast majority of mutations are essentially “private” to the specific tumor [25]. Mekenkamp et al. compared copy number aberrations in 62 primary CRC with 68 matched metastases and found a concordance rate of 88% between primary tumor and corresponding metastases. Other groups found similar results [17, 26–30]. More genetic differences were identified between primary tumors from different patients than between primary tumors and metastasis from the same patient [17, 31]. Wood et al. hypothesized that it is the overall landscape of mutations and aberrant pathways more than any single specific mutation that determines the prognosis of a patient [25].

Vermaat et al. analyzed primary CRC and matched liver metastases for mutations in genes known to be involved in cancer pathways. They found significant genetic differences, with numerous losses and gains across the genome. However, if only the routinely analyzed codon 12 and 13 KRAS mutations were considered, a concordance of >95% was found [32]. Chromosomal aberrations and mutations which are present in the primary but not in the corresponding metastases, or vice versa [26, 29], may represent either the manifestation of tumor heterogeneity and varying metastatic potential within different sub-clones, or it may represent acquisition of additional mutations during or after metastastic spread.

Sveen et al. analyzed 135 liver metastases from 45 patients to assess inter-metastatic heterogeneity, genetic complexity, and the influence of each on survival. Inter-metastatic heterogeneity was neither related to genetic complexity nor correlated with the number of metastases. Interestingly, they found that inter-metastatic heterogeneity was not only associated with poor outcome, it was an even stronger predictor of outcome than the commonly known clinic-pathological parameters in metastasized CRC [33]. This finding lends support to the suggestion by Yap et al. [13] that tumor heterogeneity may be useful as a prognostic factor.

### Mutations influencing cancer therapy

Since it became apparent that KRAS mutations are not only prognostic [34, 35] but also predictive [36, 37] of response to anti-EGFR therapy, numerous studies have investigated intra- and inter-tumor heterogeneity for KRAS status. A high concordance rate for KRAS mutation status between primary and matched distant metastases was described in most studies, ranging between 88 and 96% [38–47]. However, in two smaller study sets, the rate of discordance was much higher, ranging between 24 and 37% [48, 49].

Interestingly, the comparison of primary tumor and matched lymph node metastases revealed a much lower rate of concordance, although the level of significance was not reached in a meta-analysis [28, 45]. Testing for KRAS mutations status in lymph node metastases should therefore be avoided, if possible. The reason for this observation is not clear. Naxerova et al. [50] found that only 35% of CRC lymph node and distant metastases originate from the same subclone of the primary tumor. Distant metastases therefor seem not to sequentially arise from lymph node metastases in most cases and not necessarily share the same mutations as lymph node metatases.

The metastatic pattern of CRC has been associated with KRAS status. KRAS mutated CRC tended more often to have lung metastases compared to other sites [44, 46, 51]. This KRAS-dependent pattern of tumor spread was not found in rectal cancers [51]. Interestingly, the mutational discrepancy rate between primary tumor and lung metastases was actually higher when compared to other distant metastases (32 vs. 12%) [46].

Although KRAS status is by far the most relevant for anti-EGFR treatment, recent studies have demonstrated that tumors with other RAS, BRAF, and PIK3CA mutations also impact response to anti-EGFR treatment [52–56]. The concordance rate between primary and metastatic tumors for BRAF and PI3CA status is also high, ranging from 97 to 100% for BRAF and 93 to 95% for PI3CA [28, 41, 43, 47, 57].

Olivera et al. compared the frequency of KRAS and BRAF mutations among tumor stages in 250 primary tumors and 45 lymph node metastases. They found that KRAS and BRAF are mutually exclusive in earlier tumor stages (Tis and T1), but concomitant mutations were detectable in higher tumor stages and correlated with increased depth of invasion. Interestingly, BRAF mutations in lymph node metastases were always combined with KRAS mutations [58].

### Relevance of detection methods

The impact of the method used to identify mutations is an important practical consideration. Certain tumor sub-clones may harbor resistance to anti-EGFR therapy, and might be present in only a small fraction of the overall tumor mass, rendering them non-detectable by conventional PCR. It has been shown that these sub-clones are responsible for primary non-response or the development of secondary resistance after therapy initiation [59–62]. Molinari et al. analyzed 111 tumors for the presence of KRAS mutations, using detection methods with different sensitivity. They were able to identify 13 additional cases with KRAS mutations when using mutant-enriched PCR, which were not identified by direct sequencing. All patients with these additionally detected KRAS mutant tumors were non-responsive to anti-EGFR treatment [59]. Laurent-Puig et al. demonstrated 22 additional KRAS mutation in 136 tumors when using highly sensitive picodroplet digital PCR; these KRAS mutations were not identified by conventional qPCR. The fraction of detected KRAS mutated alleles correlated inversely with therapy response. Only patients with fewer than 1% of KRAS mutated alleles had the same outcome as patients with KRAS wild-type tumors and benefited from anti-EGFR treatment. Most resistant sub-clones can already be detected in the primary tumor, and analyzing multiple regions of the tumor with highly sensitive detection methods may allow for more personalized therapy decisions [60].

Intriguingly, sub-clones in secondarily resistant tumors exhibit a more heterogeneous pattern with several mutations in KRAS and/or other genes. These genes are mainly related to or involved in the MAPK signaling pathway [63]. This mutational pattern is unlike the pattern seen in primary resistant tumors and may be the manifestation of competitive growth in therapy-resistant sub-clones [63] by simple Darwinian selection (“survival of the fittest”).

Two exceptions exist to the rule that most mutations involved in primary and secondary resistance are concordant. First, Esposito et al. [60, 64] demonstrated that S492R mutations in the extracellular portion of the EGFR receptor were only detected in tumors exposed to cetuximab treatment. Second, Bettegowda et al. found a significant increased mutation frequency in codon 61 of KRAS and NRAS, which are very rare in untreated tumors. These two exceptions may provide evidence that tumor cells can acquire new mutations to withstand the therapy-induced selection pressure [60, 65].

## Circulating cancer cells and cell-free tumor DNA

Nowadays, tumor diagnostic can be performed on blood as well as tissue samples. Blood-based analysis is especially convenient since its collection is less invasive and does not require special equipment. In CRC patients, circulating tumor cells or cell-free tumor DNA can be examined for genetic aberrations. The literature presents conflicting statements about the concordance of RAS mutations detected in tumor tissue and in circulating cancer cells/cell-free cancer DNA. In chemotherapy- and anti-EGFR therapy-naïve patients, concordance rates of at least 93%, were reported [66, 67]. However, Pietrantonio et al. [63] reported a far lower concordance rate for several mutations, including RAS and BRAF genes in patients after developing resistance to anti-EGFR therapy. This may be the result from selecting multiple subclones with different resistance mechanisms, which could decrease the mutation load below the detection threshold of these techniques. Alternatively, this may result from variable capabilities of certain resistant sub-clones to enter the blood stream, or simply be the manifestation of sampling error in small biopsies.

Two studies sought to detect emerging resistance to anti-EGFR therapy using sequential analysis of circulating tumor DNA for KRAS status. This represents a potential non-invasive method to screen for secondary resistance due to evolving KRAS mutations. KRAS mutant DNA appeared as early as 5 to 10 months after initiation of therapy in tumors that were previously KRAS wild-type [68, 69]. Using mathematical modeling, Diaz et al. demonstrated that these new appearing mutations were likely to have been already present in a small clonal subpopulation in the primary tumor, and that the time to tumor recurrence represented the time required for the resistant sub-clone to re-populate the tumor.

Heterogeneity also exists in circulating tumor cells. They can be seen as single cells, or as clusters of 2 to 50 cells. These clusters have been shown to consist of not epithelial cells intermingled with stromal cells, and can express both stem cell and EMT markers. In breast cancer patients, tumor cell clusters were associated with a 23–50 × higher probability for metastatic spread than single circulating tumor cells. These clusters are not the result of secondary clotting in the blood stream, but are released in this form from the tumor [70, 71].

## Transcriptional heterogeneity in primary colorectal cancer and corresponding metastases

Whether a genetic mutation is actually transcribed impacts therapy response and outcome prognostication. Only a few studies have investigated the differences between RNA expression in the primary tumor and matched metastases.

Koehler et al. compared CRCs to both normal colonic mucosa and matched CRC metastases, looking for differences in mRNA expression. While marked differences were seen between normal mucosa and neoplastic cells, fewer differences were seen when primary tumors were compared with their matched metastasis. This seems to indicate that the vast majority of gene expression changes manifest in the early stages of tumorigenesis. In addition, the capability to metastasize does not rely on alterations of only a few genes, exceptionally found in metastasized tumors, rather it is the combination of different genes and the amount of gene expression alteration that fosters metastatic spread [72]. Other studies confirmed high levels of similarity between primary CRCs and matched metastases on the RNA level, and primary tumors preferentially group with their paired liver metastasis in hierarchical clustering [73, 74]. These conclusions are in line with the findings on DNA level.

Recently, microRNAs (miRNAs) have been of particular interest. MicroRNAs are small fragments of non-coding RNA that are highly involved in regulating gene expression and tumorigenesis. An elegant study by Hur et al. [75] investigated the role of the miRNA-200 family members for the metastatic process, using human samples of primary CRC, matched liver metastases, and CRC cell lines. The miRNA-200 family members are known inhibitors of epithelial-mesenchymal transition (EMT). They demonstrated a gradual decrease in miR-200c expression levels from the central part of the tumor toward tumor periphery, which can be interpreted as promotion of EMT at the tumor periphery. Additionally, miRNA-200c was found to be upregulated in CRC metastases, leading to mesenchymal-epithelial transition (MET) and supporting the role of these regulators in metastatic spread. This upregulation of miRNA led to downregulation of its target genes—ZEB1, ETS and FLT1—which in turn led to upregulation of e-cadherin and downregulation of vimentin, two prominent proteins affected by EMT and MET. Remarkably, the expression of miR-200 was found to be regulated by methylation of its promoter region. This potentially reversible epigenetic regulation may permit alternating switches between EMT and MET. Diaz et al. [76] demonstrated that high levels of miR-200a and miR-200c in primary tumors are associated with prolonged overall survival. In contrast, other studies have demonstrated that miR-200 family members are significantly downregulated in liver metastases [77, 78].

Numerous other microRNAs, such as miR-196b-5p, miR-320b, miR-320d, and miR-429, have also been found to exhibit differential expression when primary tumors are compared to their corresponding metastases [78–80].

## Translational heterogeneity in primary colorectal cancer and corresponding metastases

Few studies compare protein expression in primary colorectal cancer and matched metastases. Zhang et al. illustrated that copy number alterations have a direct influence of the abundance of mRNA, but the amount of protein expression cannot be reliably predicted based on genetic alterations or mRNA levels [81].

## Is it all about tumor cells?—the role of the microenvironment

What would a tumor be without its microenvironment? The role of tumor microenvironment on tumorigenesis and tumor progression has seen considerable interest. It remains unclear to what extent the microenvironment is involved in tumor development, and to what extent it may differ from tumor to tumor. However, it is clear that the microenvironment influences gene expression in cancer cells, and is a significant factor contributing to tumor heterogeneity. This is true for intra-tumor heterogeneity, and especially true for inter-tumor heterogeneity, as the microenvironment of distant sites can differ considerably from the primary site.

Pennacchietti et al. showed that microenvironment heterogeneity directly influences heterogeneity of cancer cells [82]. They demonstrated that hypoxia leads to overexpression of hepatocyte growth factor, resulting in tumor cell migration and invasion in cell culture. Tumor buds, single cells or small clusters of tumor cells at the invasion front of the main tumor, have been claimed to epitomize EMT. Decreased expression of E-Cadherin, miR-200b, and miR-200c as well as upregulation of EMT markers in tumor buds support this concept [83]. Vermeulen et al. showed β-catenin-dependent expression of cancer stem cell features due to secretion of myofibroblast-derived factors such as hepatocyte growth factor. This study also demonstrated that the cancer stem cell concept is more dynamic than assumed, as more differentiated cells were able to restore stem cell features due to activation of the Wnt-pathway [84].

Microenvironment contributions to tumor development can be easily recognizable on H&E stain, including vascularization, infiltrating tumor-associated inflammatory cells, or stromal cells [24, 85]. Taken together, this implies that tumor heterogeneity is not entirely derived from genetic, translational, or transcriptional changes within the tumor cell alone, but that heterogeneity is also partly contributed by the tumor microenvironment.

## Conclusions

Tumor heterogeneity is accepted fact, and heterogeneity seems particularly pronounced in CRC. Heterogeneity is not confined to the genetic level, but also manifests with epigenetic changes, and the tumor microenvironment. The presence of tumor heterogeneity is of considerable clinical interest, as it directly impacts treatment decisions. Small biopsies, for instance, harbor an intrinsic risk of sampling error when tissue is tested for therapy-related biomarkers such as KRAS, which may not be uniform in all sub-clones [5, 14, 16]. The presence of sub-clones that may fall below certain detection thresholds makes the choice of testing method clinically significant. Highly sensitive detection methods should be preferred to avoid false-negative results, as even small fractions of KRAS mutated tumor cells have been shown to permit the development of secondary-resistance shortly after treatment initiation. Liquid biopsies may be useful in identifying secondarily resistant sub-clones even before clinically apparent tumor relapse.

Although the apple does not fall far from the tree in colorectal cancer, tumor heterogeneity must be recognized and considered in the clinical treatment paradigm.

## Author contributions

AB: writing and editing of the original draft; DR, HD, and IZ: reviewing and editing; AL: writing, reviewing, and editing.

### Conflict of interest statement

The authors declare that the research was conducted in the absence of any commercial or financial relationships that could be construed as a potential conflict of interest.
